# Ovicidal Activity of Organophosphate, Oxadiazine, Neonicotinoid and Insect Growth Regulator Chemistries on Northern Strain Plum Curculio, *Conotrachelus nenuphar*

**DOI:** 10.1673/031.008.2901

**Published:** 2008-04-09

**Authors:** Eric J. Hoffmann, Samantha M. Middleton, John C. Wise

**Affiliations:** Department of Entomology, Michigan State University, 243 Natural Science, East Lansing MI 48824

**Keywords:** *in vitro* assay, partitioning coefficient, curative activity

## Abstract

An *in vitro* method was developed for assessing ovicidal effects of the organophosphate azinphos-methyl, the neonicotioids thiacloprid, thiamethoxam and clothianidin, the oxadiazine indoxacarb and the insect growth regulators novaluron and pyriproxifen on the plum curculio, *Conotrachelus nenuphar* (Herbst)(Coleoptera: Curculionidae). The baseline survivorship of this method was 88 percent. Plum curculio eggs were most sensitive to azinphos-methyl. Thiacloprid, clothianidin and the chitin synthesis inhibitor, novaluron, had LC50 values below 100 ppm. Thiamethoxam, indoxacarb and pyriproxifen were not ovicidal at 100 ppm. Octanol-water partitioning coefficients, log *Kow,* appeared to be an important indicator of ovicidal activity within the neonicotinoids. This new bioassay method eliminates the confounding of the insect-chemical and plant-chemical interactions and the results highlight the utility of a post-infestation curative approach to plum curculio management.

## Introduction

The plum curculio, *Conotrachelus nenuphar* (Herbst)(Coleoptera: Curculionidae), is an endemic pest of tree fruit in Eastern North America. The northern strain of this insect is univoltine, with an obligate adult overwintering diapause. The southern strain has a facultative diapause. Both strains are serious pests of cultivated stone and pome fruits (Quaintance and Jenne 1912; Hallman and Gould 2004).

For apples grown in Michigan and New York and other Great Lakes States, oviposition by *C. nenuphar* occurs in the 6 – 10 weeks (400 Growing Degree Days 10°C – GDD10°C) after petal fall (Reissig et al. 1998). Eggs are laid just underneath the fruit skin after the female makes a small feeding incision. After oviposition, the female also chews a C-shaped excavation around the egg, which is thought to prevent local tissue expansion and protect the egg from being subsequently crushed (Owens et al. 1982). Eggs take 3–6 days to hatch (Smith 1957; Mampe and Neunzig 1967) and larvae are exclusively internal feeders. Whether or not the eggs hatch, the oviposition incision develops into a surface scar and can render fruit unacceptable for fresh markets. Larval presence inside of fruit is a key regulatory concern for processed commodities like tart cherries, where there are zero-tolerance standards in place for insect infestation (USDA Agricultural Marketing Service 1941a,b).

The management of this pest is overwhelmingly focused on control of adults (Smith 1964; Howitt 1993; Ressig et al. 1998). Organophosphorus insecticides (primarily azinphos-methyl) are currently the primary means of *C. nenuphar* control, but newer classes are being aggressively studied in light of the FQPA-directed phaseout of the organophosphate azinphos-methyl (US EPA 2006). These new classes (neonicotinoids, oxadiazines, and insect growth regulators) generally lack the acute adult contact toxicity of the organophosphorus compounds and require close examination to fully understand their potential uses in *C. nenuphar* management.

Post-infestation, or curative, action is one of the possible modes of activity for chemical control. The early organophosphates parathion and EPN [*O*-ethyl *O*-(*p*-nitrophenyl) phenylphosphonothioate] were identified as having some ovicidal and larvicidal activity against *C. nenuphar* (Smith et al. 1956), but this was primarily viewed as a secondary benefit of these adult-targeted materials. Currently- registered organophosphate and neonicotinoid insecticide sprays have also been shown to penetrate into apple fruit tissue at concentrations sufficient to kill the internally-feeding *C. nenuphar* larvae (Wise et al. 2007).

Insect growth regulators also show lethal activity when applied to eggs of certain insect species. The chitin synthesis inhibitor diflubenzuron killed eggs of the codling moth (Charmillot et al. 2001), and teflubenzuron and hexaflumuron are effective against eggs of the cowpea weevil (Abo-Elghar et al. 2003). The juvenile hormone analog pyriproxifen was ovicidal when applied to eggs of codling moth (Charmillot et al. 2001; Yokoyama and Miller 1991), diamondback moth (Oouchi 2005), and whiteflies (Isaaya et al. 1994). The effectiveness of this class against *C. nenuphar* eggs has not been studied.

The current study examined the toxicity of current crop protection compounds to *C. nenuphar* eggs. The challenge of regulating chemical concentrations in the fruit required the development of an *in vitro* assay that truly isolated the insect-chemical interaction from other influences like varying chemical penetration and movement through plant tissues and plant metabolism of the insecticide compounds.

## Materials and Methods

### Insect Source and Maintenance

Northern strain *C. nenuphar* were collected from 5 May – 10 June 2006 in cherry and apple orchards at the Trevor Nichols Research Complex in Fennville, MI (42.5951°N, -86.1561°W) using commercially-available pyramid traps (Tedders and Wood 1994) and a pneumatic limb shaker (Maibo Model ST-7-06, distributed by Tree Tools LLC, www.treetools.com/catalog/quadel). Weevils were sexed according to the method of Thomson (1932) and placed into gender-separate screen cages (Model 1450 B BioQuip Products Inc., www.BioQuip.com) after a 2 week mating period. Beetles were provided with untreated cherry branches (*Prunus cerasus* var. Montmorency) with fruit and foliage in wetted floral foam (OASIS Smithers-Oasis Co. www.smithersoasis.com. When preparing to harvest a unified cohort of eggs, females were provided fresh, undamaged fruit for 24h.

### Chemicals

A well-plate *in* *vitro* method was used to determine the ovicidal toxicity of azinphos-methyl (Guthion 50W, Bayer CropScience, www.bayercropscience.com), thiamethoxam (Actara 25WG, Syngenta, www.syngenta.com), thiacloprid (Calypso™ 4F, Bayer CropScience), clothianidin (Clutch™ 50 WDG, Arysta LifeScience, www.arysta-na.com), indoxacarb (Avaunt 30 WG, DuPont, www.dupont.com), novaluron (Rimon 0.83 EC, Chemtura Corporation, www.chemtura.com), and pyriproxifen (Esteem 35WP, Valent Agricultural Products, www.valent.com). Formulated materials were prepared in distilled water with 0.125% (by volume) Latron B-1956 (Rhom and Haas, www.rohmhaas.com) as a surfactant; control treatments were water and surfactant only. Initial survey concentrations were prepared at 100, 10, and 1.0 and 0.1 ppm AI. Initial survey activity (if any) informed the concentrations used for secondary screening for LC50 calculations.

### Egg Bioassay

The egg toxicity assays were set-up in 96 well cell plates (Corning Inc., Corning, NY). Every other perimeter cell of the plate had 300 µl of distilled water to minimize desiccation within the experimental cells. This plate setup allowed for six treatments (plus a control) of 10 cells per treatment. A 4mm × 8mm square of Whatman #1 filter paper was inserted in each of the interior wells, along with 30 µl of chemical solution (or water control). This initial amount was sufficient to keep eggs hydrated through the incubation period without the need for additions. A set of 8–10 control eggs per replicate was used to correct for method mortality and variations in incubation conditions.

**Figure 1.**
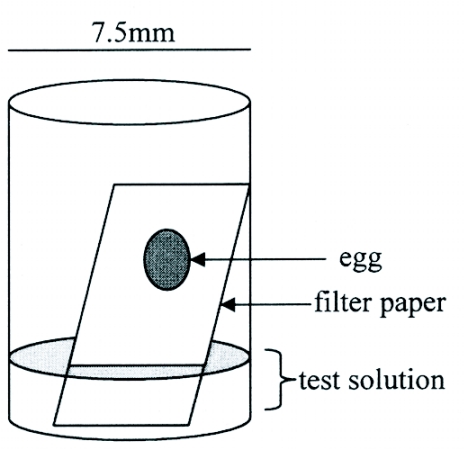
Single well of the egg bioassay.

Eggs were harvested from the fruit using a needle-like probe, forceps, and a dissecting microscope (Model 47 50 61 Carl Zeiss Inc., www.zeiss.com), and placed on the filter paper 2–4 mm above the solution level, one egg per well (Figure 1). If eggs ruptured in transfer, a new paper was placed in the well. Although eggs were not directly in contact with the liquid, wicking action of the filter paper was sufficient to keep the eggs hydrated throughout the incubation period (about 5 days for untreated eggs). Plates were kept at 22 ± 4°C and 16:8 L:D. Hatched larvae were recorded and removed daily for 10 days.

### Data Analysis

Egg hatch data were adjusted for untreated mortality (Abbott 1925), and LC50 values were calculated using PROC PROBIT in SAS (SAS Institute 2000). Confidence limits and slopes of regression lines were also derived from this procedure.

## Results

The 96-well plate *in vitro* method was an effective way to incubate eggs. The hatching percentage of the control cells was 87.7 ± 12.0 (*N* = 225). Egg desiccation was not observed in the controls and normal hatch began 5 days after females were first provided cherries for oviposition. *C. nenuphar* eggs have a relatively soft chorion, and egg rupture during harvesting and transfer was not uncommon. Sharpened forceps were appropriate for peeling back the fruit skin, but blunted metal probes (14 mm length, tapering to 0.25 mm tip) worked best for the removal of the egg and transfer to the filter paper.

Azinphos-methyl and novaluron were the most toxic to *C. nenuphar* eggs of the screened compounds, although eggs were much more sensitive to azinphos-methyl (Table 1). Activities of the neonicotinoids, thiacloprid and clothianidin, were similar, but thiamethoxam was not active against *C. nenuphar* eggs. Neither indoxacarb, an oxadiazine, nor pyriproxifen, an insect growth regulator, reduced egg hatch at the concentrations used.

**Table 1.**
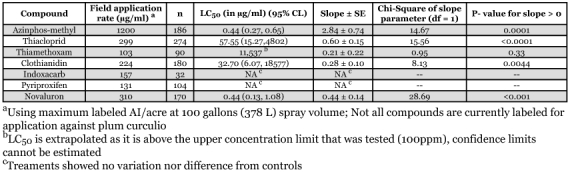
Toxicity profiles for seven compounds applied to plum curculio, *Conotrachelus nenuphar,* eggs. Mortality was determined 10 days after incubation.

## Discussion

An efficient *in vitro* ovicidal assay is an important tool for evaluating new insecticides for the control of *C. nenuphar.* Mortality of controls was low, and it is a robust screening technology for *C. nenuphar.* The well-plate method would be appropriate for any system where eggs are laid inside of plant tissue and can be extracted without damaging the developing embryos. With this technique, field-based efficacy data, residue analyses and baseline toxicity data can be linked to more completely evaluate the potential for targeting eggs with insecticides.

Application and residue data from field applications are required to put these *in vitro* data into context. Labeled application rates for these compounds are shown in Table 1. After a field-rate foliar spray, azinphos-methyl was recovered from the outer 2 mm of apple flesh at 1.76 ppm, and this dosage significantly reduced larval emergence from fruit treated after egg hatch (Wise et al. 2007). The LD50 for azinphos-methyl exposure to *C. nenuphar* adults was 160 ppm (Wise et al. 2007). The LC95 for azinphos-methyl in the current ovicidal study was 1.68 ppm. Collectively, these life-stage specific studies suggest that azinphos-methyl performance is likely achieved through a combination of adult, egg, and larval activity. Wise et al. (2007) recovered 0.01 ppm thiacloprid and 0.05 ppm novaluron from the outer 2 mm of apple flesh after treatment with labeled rates of these compounds. These recoveries are markedly less than the LC50 concentrations demonstrated for eggs. Thiacloprid did show a curative effect in larval-targeted field-based applications, but no effect was observed with novaluron applications to infested apples (Wise et al. 2007). Susceptibility to these compounds clearly depends on the exposed life stage.

It should be noted that insecticide residues inside fruit that would act as ovicides are transient, and occur early in the season relative to harvest. The reported residues in penetration studies are a result of labeled application protocols, and harvested materials meet the legal thresholds for insecticide residue concentrations.

Despite sharing the same target site and mode of action, the variation in ovicidal action among the tested neonicotinoids is striking. Ovicidal activity of this class against *C. nenuphar* correlates well with the octanol-water partitioning coefficient (log *Kow*) of these compounds. Since the lipid layers of the insect chorion provide a general barrier to hydrophilic (low- log *Kow*) materials (Smith and Salkeld 1966), compounds like thiamethoxam (log *Kow* = -0.13) are unlikely to reach target sites within the embryo. Thiacloprid and clothianidin both have positive partitioning coefficients and are therefore better able to move through the chorion. Thiamethoxam is a precursor to clothianidin, and is converted to clothianidin in both plants and insects (Nauen et al. 2003). Foliar application of thiamethoxam may provide both a surface residue profile of the parent compound, as well as ovicidal activity by the conversion product after it has penetrated into the plant tissue. Formulated clothianidin is not currently labeled for use in cherry orchards.

The variable ovicidal activity profile across neonicotinoids has been noted in other studies as well, and generally follows the patterns described above. Acetamiprid (log *Kow* = 0.8) was highly effective against bollworm eggs, while thiamethoxam and imidacloprid (log *Kow* = 0.57) both showed less activity (Kilpatrick et al. 2005). In the multicolored Asian lady beetles, acetamiprid and imidacloprid were both highly toxic to eggs while thiamethoxam had no significant effect (Youn et al. 2003).

However, partitioning coefficients are not absolute predictors of activity. The oxadiazine indoxacarb is highly lipophilic, but is completely inactive against *C. nenuphar* eggs. This compound is primarily an ingestion-active material (Wing et al. 2000), so it is not surprising that it does not work against the embryonic stage.

Comprehensive control of *C. nenuphar* in the absence of organophosphates will likely require a suite of tactics and life-stage targets. Although adult control during the growing season will likely remain the mainstay, investigation of alternative avenues are needed to completely understand the impact of field treatments on curculio populations. Curative activity represents one such approach, but it is not appropriate for all of the crops that are susceptible to *C. nenuphar* damage. Fresh market commodities must meet high consumer quality demands and oviposition scarring is not acceptable for many consumers. However, processed markets (juices, canned and frozen fruits) do not have these aesthetic concerns. A curative approach would allow these crops to meet the principal mandate of infestation-free fruit.
